# Epac1^−/−^ mice have elevated baseline permeability and do not respond to histamine as measured with dynamic contrast‐enhanced magnetic resonance imaging with contrast agents of different molecular weights

**DOI:** 10.1111/apha.13199

**Published:** 2018-11-02

**Authors:** Fitz‐Roy E. Curry, Torfinn Taxt, Cecilie Brekke Rygh, Tina Pavlin, Ronja Bjønrstad, Stein Ove Døskeland, Rolf K. Reed

**Affiliations:** ^1^ Department of Physiology and Membrane Biology University of California Davis Davis California; ^2^ Department of Biomedicine University of Bergen Bergen Norway; ^3^ Molecular Imaging Centre Department of Biomedicine University of Bergen Bergen Norway; ^4^ Centre for Cancer Biomarkers University of Bergen Bergen Norway; ^5^ Present address: Western Norway University of Applied Sciences Bergen Norway; ^6^ Present address: NordicNeurolab AS Bergen Norway; ^7^ Present address: Department of Clinical Science University of Bergen Bergen Norway

**Keywords:** capillary permeability, compartment model, deconvolution, Dotarem, Epac1, histamine

## Abstract

**Aim:**

Epac1^−/−^ mice, but not Epac2^−/−^ mice have elevated baseline permeability to albumin. This study extends the investigations of how Epac‐dependent pathways modulate transvascular exchange in response to the classical inflammatory agent histamine. It also evaluates the limitations of models of blood‐to‐tissue exchange in transgenic mice in DCE‐MRI measurements.

**Methods:**

We measured DCE‐MRI signal intensity in masseter muscle of wt and Epac1^−/−^ mice with established approaches from capillary physiology to determine how changes in blood flow and vascular permeability contribute to overall changes of microvascular flux. We used two tracers, the high molecular weight tracer (Gadomer‐17, MW 17 kDa, apparent MW 30‐35 kDa) is expected to be primarily limited by diffusion and therefore less dependent on changes in blood flow and the low molecular weight tracer (Dotarem (MW 0.56 kDa) whose transvascular exchange is determined by both blood flow and permeability. Paired experiments in each animal combined with analytical methods provided an internally consistent description of microvascular transport.

**Results:**

Epac1^−/−^ mice had elevated baseline permeability relative to wt control mice for Dotarem and Gadomer‐17. In contrast to wt mice, Epac1^−/−^ mice failed to increase transvascular permeability in response to histamine. Dotarem underestimated blood flow and vascular volume and Gadomer‐17 has limited sensitivity in extravascular accumulation.

**Conclusion:**

The study suggests that the normal barrier loosening effect of histamine in venular microvessels do not function when the normal barrier tightening effect of Epac1 is already compromised. The study also demonstrated that the numerical analysis of DCE‐MRI data with tracers of different molecular weight has significant limitations.

## 1 INTRODUCTION

Investigations in cultured endothelial cell monolayers and intact microvessels have demonstrated that cAMP dependent Epac1 pathways modulate acute inflammatory responses in endothelial barriers and exert a tonic action to maintain normal baseline permeability.^1^, ^2^ However, the contribution of these pathways to vascular permeability regulation in intact microvascular beds is less well understood. The availability of mice with knockout of Epac1 and Epac2 has enabled us to study the role of the Epac pathways in the intact microcirculation where the effects of Epac pathways on both permeability and blood flow need to be evaluated. We recently reported that Epac1^−/−^ mice, but not Epac2^−/−^ mice, had elevated baseline permeability to albumin.^3^ Furthermore, atrial natriuretic peptide (ANP), a physiological modulator of vascular permeability, increases the vascular permeability to albumin in wild‐type (wt) mice as well as Epac2^−/−^ mice. In contrast, the Epac1^−/−^ mice did not increase their permeability in response to ANP above the already increased baseline.^3^

The primary aim of the present study was to use Epac1^−/−^ mice to evaluate the role of Epac‐dependent pathways in the modulation of transvascular exchange in response to the classical inflammatory agent histamine. Although genetically modified mice offer a wealth of opportunities for extending advances in molecular and cellular biology at the organ and organism level, there have been few detailed investigations of microvascular exchange, and in particular, the importance of plasma flow and permeability in overall microvascular transport using such animals. Some of the reasons for this include the novel technical challenge of adjusting procedures and detection sensitivity to the small animal size. Another is the relative hyperdynamic circulation compared to larger animals.^4^

Thus, a second aim of the present study was to explore these physiological problems by extending previous methods using genetically modified mice with increased capillary permeability and to use dynamic contrast‐enhanced magnetic resonance imaging (DCE‐MRI).^3^, ^5^ We used two tracers with different molecular weight to measure tissue uptake under conditions where blood flow and vascular permeability were increased by histamine. The transport of the high molecular weight tracer Gadomer‐17 (MW 17 kDa and apparent MW 30‐35 kDa) across the microvascular wall is expected to be primarily limited by diffusion in mouse masseter muscle and therefore less dependent of changes in blood flow. The lower molecular weight tracer was Dotarem (MW 0.56 kDa) for which the exchange is determined by both blood flow and permeability.^6^

We have evaluated numerical methods to account for rapid changes in the arterial tracer concentration (so‐called blind deconvolution) and compartmental models that describe both diffusion limited and flow limited exchange, as well as exploit the fact that DCE‐MRI enables repeated measurements days apart using tracers of varying molecular weights. The DCE‐MRI is combined with numerical analysis as described in Methods using the Johnson‐Wilson model for capillary exchange^7^ based on the classical Kety/Renkin/Crone model of tracer extraction in a perfused microvessel as well as a compartmental model where the ratio of local vascular volume and exchange area is assumed to remain fixed as blood flow changes.

The main conclusion from the present study is that Epac1^−/−^ mice have elevated baseline permeability relative to wt (C57black) control mice to Dotarem as well as Gadomer‐17. In contrast to wt mice, Epac1^−/−^ mice failed to increase their microvascular permeability in response to histamine. This suggests that the barrier loosening effect of Epac1^−/−^is already at the maximum that histamine would cause in wt mice. The study demonstrates that paired sequential experiments using more than one tracer in each animal combined with analytical methods provide a more internally consistent description of modulations of microvascular function than experiments using only one tracer and unpaired experimental design. Finally, the study also demonstrated that the numerical analysis of DCE‐MRI data with tracers of different molecular weight has significant limitations.

## 2 RESULTS

### 2.1 Overview of data and analyses of tracer signal intensity curves in mice

**Figure 1:**
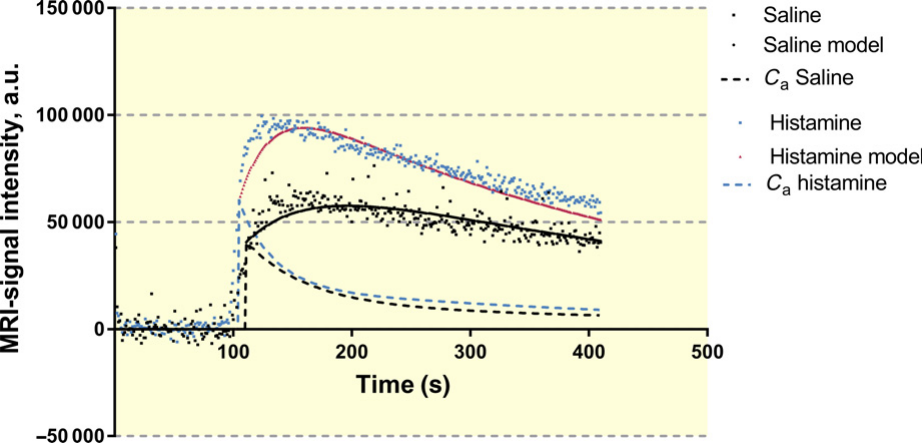
MRI signal intensity in control (i.v. saline) and after histamine administration represented as black and blue squares respectively. Also shown are results when using the two compartment model for tissue concentration (solid line) and in plasma (*C*_a_, dashed line) with black and red representing saline and histamine administration respectively

Before we describe the results that address changes in vascular permeability, surface area for exchange and blood flow in response to histamine in wt and Epac1^−/−^ mice, we first describe the general form of the signal intensity (SI) curves from the contrast‐enhanced MRI measurements used to obtain these exchange parameters. For all experiments, we placed a region‐of‐interest (ROI) over the masseter muscle away from any large blood vessel to fulfil the assumptions in the model that the SI signal was mainly determined by exchange in small microvessels with diameter of 5‐15 μm. Figure 1 shows the SI curves from paired experiments in a wt mouse where the low molecular weight MRI contrast agent Dotarem (MW 0.56 Kd) was injected via the tail vein 1.5 minutes after saline (control) (black squares) and then, 24 hours later, 1.5 minutes after histamine (blue squares). The shape of SI curves was the same for all experiments. With a bolus injection of tracer (176 μL within 15 seconds), there was an initial step increase in SI followed by a further SI increase as additional tracer crossed the vascular wall and accumulated in the tissue. SI continued to increase because the concentration difference between plasma and the extravascular space favoured plasma‐to‐tissue diffusion. Plasma tracer concentrations fell as Dotarem diffused into all the mouse tissues and was excreted by filtration in the kidney. Tracer accumulation in the tissue reached a peak when the fall of vascular tracer concentration was sufficient for the tracer to begin to diffuse back out of the tissue compartment.

### 2.2 Fitting Signal Intensity (SI) curves for Dotarem and Gadomer‐17 using the Compartmental Model

Figure 1 shows that the time to complete a step increase in tracer concentration was at least 20 seconds (up to 3 circulation times in the mouse). The curves in Figure 1 were obtained by integrating the expression for Fick's Law of Diffusion as described in the Methods taking into account the fall in vascular tracer concentration. Model curves that best described the entire time course of the tracer were used to evaluate a characteristic time constant for tracer exchange between plasma and tissue (PS/V_a_) where PS is the permeability‐surface area product for the tracer and *V*_a_ is the local vascular volume.

The fall in vascular tracer concentration within the measuring window was described by two time constants as outlined in Methods. One accounted for equilibration of tracer into all the tissues of the body and the other for renal excretion of the tracer. In Figure 1, the half‐time for the vascular tracer (broken black curve) was 37 seconds for control experiments and 23 seconds after histamine (broken blue curve). These values were consistent with independent estimates of the half‐time of intravascular Dotarem in wt mice using the deconvolution analysis (see Tables 1 and 2). As noted in Methods, the second rate constant was included to account for renal excretion of Dotarem by glomerular filtration based on measurement of glomerular filtration rate. Renal excretion was not included for the larger Gadomer‐17 tracer. It is noted that there was always some discrepancy between the model prediction and the measured tissue SI during the first 20‐30 seconds after injecting the tracer, reflecting, at least in part, the limitation of describing the initial vascular filling as a step increase in tracer amount.

**Table 1:** Effect of histamine in wild‐type and Epac1^−/−^ mice using Dotarem and studied by DCE‐MRI (MW 0.56 kDa). The results are based on repeated measurements, i.e. after control measurements with i.v. saline a new measurement was performed 1 day later using i.v. histamine administration. The Johnson‐Wilson deconvolution model was used for calculation. The confidence of blood flow and blood volume are evaluated in Discussion

	Wild type_(n = 7)		Epac1^−/−^ (n = 10)	
	Saline	Histamine	Saline	Histamine
Primary derived parameters				
Blood flow (*F*_b_), mL 100 mL^−1^ min^−1^	6.27 ± 1.16	11.81 ± 1.64***	6.81 ± 1.06	9.1 ± 3.64
Efflux rate from *V*_e_, to *V*_b_ (*k*_ep_), min^−1^	0.443 ± 0.087	0.691 ± 0.074***	0.555 ± 0.071##	0.478 ± 0.148*
Extraction fraction	0.739 ± 0.053	0.516 ± 0.037***	0.715 ± 0.047	0.590 ± 0.081** ^,^ #
Intravascular transit time (*T*_c_), seconds	17.6 ± 3.05	8.79 ± 2.02***	14.0 ± 3.64	11.6 ± 2.39^(^ # ^)^
Plasma *t*_1/2_, seconds	36.7 ± 8.2	22.6 ± 17.1***	25.9 ± 6.0#	21.1 ± 5.2*
Secondary derived parameters				
Blood volume (*V*_b_), mL 100 mL^−1^	1.82 ± 0.38	1.70 ± 0.27	1.58 ± 0.41	1.70 ± 0.70
PS, mL 100 mL^−1^ min^−1^	6.08 ± 1.21	6.16 ± 0.49	6.11 ± 0.42	5.83 ± 2.72
Extravascular space (*V*_e_), mL 100 mL^−1^	10.44 ± 1.45	8.85 ± 0.89***	8.81 ± 1.35#	11.7 ± 6.30#
Transfer constant *V*_b_ to *V*_e_ (*k*_trans_), min^−1^	3.32 ± 0.60	4.36 ± 0.41***	3.48 ± 0.38	3.80 ± 1.59

Mean ± SD. *P < 0.05, **P < 0.01 and ***P < 0.001 histamine vs control; ^#^ *P* < 0.05, ^##^ *P* < 0.01 same condition comparing wt vs Epac1^−/−^. ^(#)^ *P* = 0.052.

**Table 2:** Comparison of Dotarem (MW 0.56 kDa) and Gadomer‐17 (MW 17 kDa and apparent MW 30‐35 kDa) in six wt mice where Dotarem was injected first and Gadomer‐17 was injected 1 day later. Johnson‐Wilson deconvolution method was used for calculation in these experiments. Intravascular transit times (*T*_c_) from the Dotarem were used in the Gadomer‐17 experiments in order for the model to converge and provide a solution. The confidence in the reported values of blood flow and volume for Dotarem and Extraction reaction for Gadomer‐17 are evaluated in Discussion

	Dotarem	Gadomer‐17
Primary derived parameters		
Blood flow (*F*_b_), mL 100 mL^−1^ min^−1^	7.47 ± 1.84	16.60 ± 7.80*
Intravascular transit time (*T*_c_), seconds	11.7 ± 3.00	11.7 ± 3.00^cf legend^
Extraction fraction	0.657 ± 0.043	0.074 ± 0.027***
Efflux rate from *V*_e_, to *V*_b_ (*k*_ep_), min^−1^	0.613 ± 0.109	0.225 ± 0.078***
Plasma t_1/2_, seconds	20 ± 4	637 ± 507*
Secondary derived parameters		
Blood volume (*V*_b_), mL 100 mL^−1^	1.39 ± 0.26	2.94 ± 0.81**
PS, mL 100 mL^−1^ min^−1^	5.67 ± 0.90	0.83 ± 0.28***
Extravascular volume (*V*_e_), mL 100 mL^−1^	7.98 ± 1.30	4.96 ± 0.95**
Transfer constant *V*_b_ to *V*_e_ (*k*_trans_), min^−1^	3.49 ± 0.68	0.79 ± 0.27***

Mean ± SD. *P < 0.05, **P < 0.01 and ***P < 0.001 for Dotarem vs Gadomer‐17.

The use of the compartmental model to analyse SI curves using the larger Gadomer 17 as the tracer was much simpler because the time constants for tracer fall were found to be more than ten times longer than for Dotarem and tracer accumulation in tissue increased more slowly.

### 2.3 Fitting SI curves for Dotarem and Gadomer‐17 using the Johnson‐Wilson model and deconvolution

An unexpected result was that we had far more problems with the analysis based on the Johnson‐Wilson model and the advanced curve fitting (deconvolution) analysis than with the compartmental model. As described in the Methods, we first estimated the four primary model parameters: intravascular transit time *T*_c_, plasma flow *F*_p_, extraction fraction *E* and *k*_ep_, a tissue to plasma rate constant for exchange. The values of the plasma volume *V*_a_, extravascular volume *V*_e_ and permeability‐surface area product PS are derived from these directly estimated parameters and are therefore not independent parameters. An important result indicating limitations of this approach was that estimates of *F*_p_ and *V*_a_ using Dotarem and Gadomer‐17 were significantly different (see Tables 1 and 2 below where plasma flows are reported as equivalent blood flows). These values should have been the same for the two tracers under the same perfusion conditions. Furthermore, Gadomer‐17 extraction fractions fell below 0.1 in contrast to Dotarem (extraction fraction >0.5). This limited the sensitivity of the Gadomer‐17 to the changes in extravascular tracer accumulation. However, because Gadomer‐17 was to a larger extent retained in the vascular space, it was a more reliable vascular tracer. In the legends to Tables 1 and 2, we have indicated the parameters whose values are least reliable. In spite of these limitations, we have demonstrated a clear difference in the response of Epac1^−/−^ mice to histamine compared to wt controls as described below.

### 2.4 Response to histamine in wt and Epac1^−/−^ mice: compartmental analysis using Dotarem

**Figure 2:**
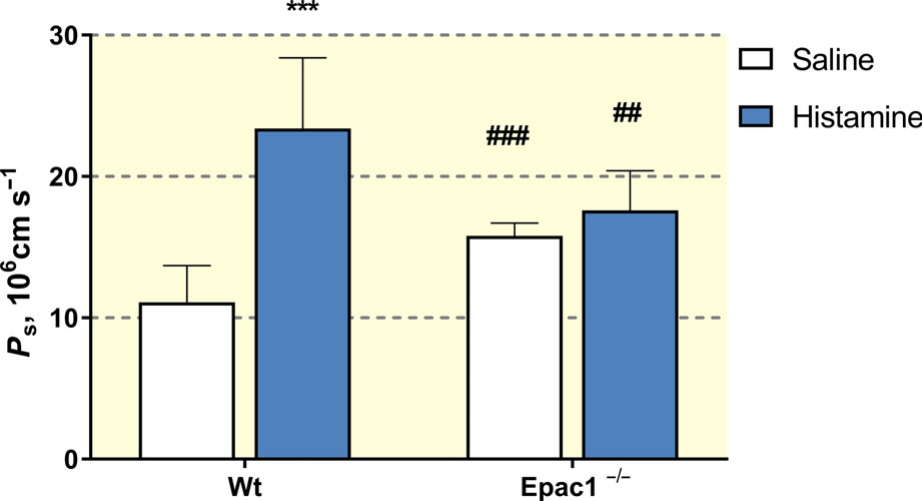
Permeability (*P*_s_) for Dotarem in wild‐type (wt) mice and Epac1^−/−^ mice in control (i.v. saline) and after histamine administration determined with the two compartment model. ***P < 0.001 for histamine vs control. ^##^ *P* < 0.01 and ^###^ *P* < 0.001 for the same situation comparing between wt and Epac1^−/−^

The primary rate constant from the analysis is the ratio PS/*V*_a_ where *V*_a_ is the local plasma volume. In wt mice, PS/*V*_a_ increased from 0.025 ± 0.006 s^−1^ to 0.053 ± 0.011 s^−1^ (*P* < 0.001) with histamine. In Epac1^−/−^ mice, the baseline value of PS/*V*_a_ was 0.036 ± 0.007 s^−1^ and was significantly increased compared to wt saline controls but histamine did not increase PS/*V*_a_ further (0.040 ± 0.006 s^−1^). Figure 2 summarizes the estimates of the permeability coefficients (*P*_s_) calculated from the mean rate constants from the paired experiments (saline control vs histamine) for Dotarem in wt and Epac1^−/−^ mice. The permeability coefficients in Figure 2 are calculated assuming an average value for the ratio of vascular volume to surface area (*V*_a_/*S*) of 4.4 × 10^−4^ cm as described in Methods. According to this analysis, histamine increased the Dotarem permeability coefficient in wt mice from 11.1 ± 2.6 to 23.5 ± 4.7 × 10^−6 ^cm s^−1^ (*P* < 0.001). The baseline permeability to Dotarem in Epac1^−/−^ animal in control was 15.8 ± 0.9 10^−6^ compared to 11.1 ± 2.6 × 10^−6^ cm s^−1^ in wt (*P* < 0.01). However, in Epac1^−/−^ mice, histamine did not significantly increase the permeability coefficients to Dotarem (17.6 ± 2.6 × 10^−6^ cm s^−1^ vs the elevated baseline of 15.8 ± 0.9 × 10^−6^ cm s^−1^).

### 2.5 Wild type vs Epac1^−/−^ using Gadomer‐17 and the two‐compartmental model

**Figure 3:**
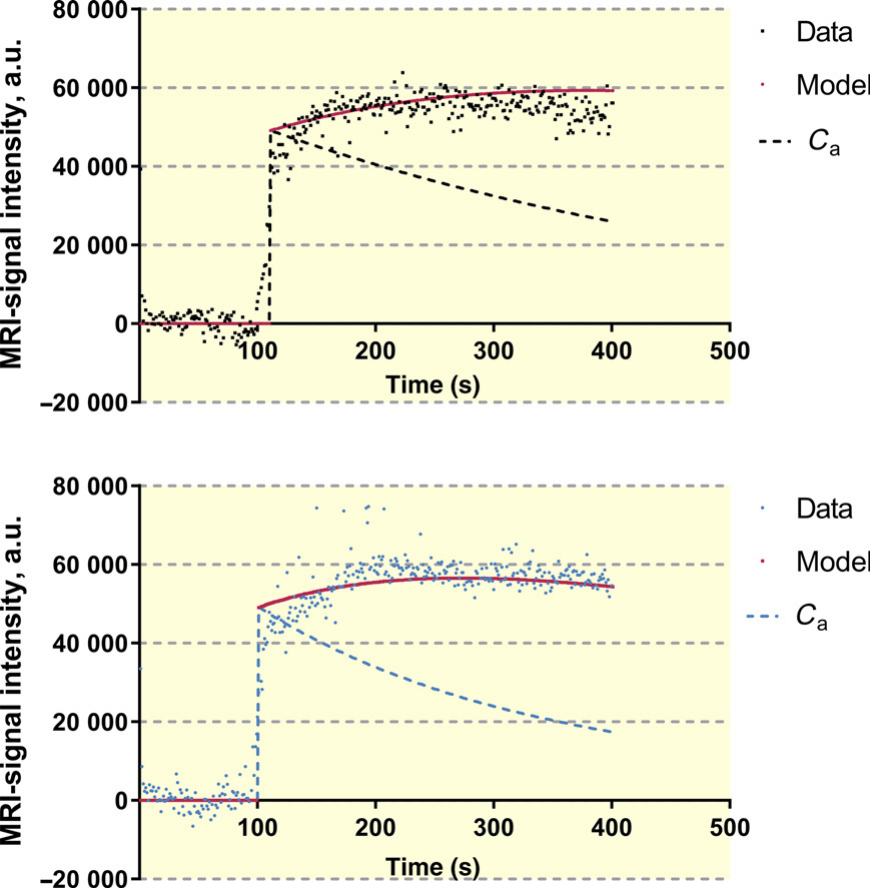
A (Upper panel), Recording of MRI‐signal (black circles) after Gadomer‐17 in control (i.v. saline) and analysed with the two compartment model. Solid and dashed lines represent modelling of tissue data and arterial concentration (*C*_a_) respectively. B (Lower panel), Recording of MRI‐signal recording (blue circles) after Gadomer‐17 and histamine administration and analysed with the two compartment model. Solid and dashed lines represent modelling of tissue data and arterial concentration (*C*_a_) respectively

Figures 3A,B show one example to fit paired measurements of the tissue uptake curve for Gadomer‐17 with and without histamine in a wt mouse. The mean half‐times for the fall in arterial concentration were 339 s^−1^ with saline and 190 s^−1^ after histamine administration. This was determined by the requirement to simultaneously fit the initial tracer accumulation after Gadomer‐17 filling of the vascular space and the time after the total tissue tracer accumulation began to fall as the Gadomer‐17 equilibrated with other tissues in the body.

**Figure 4:**
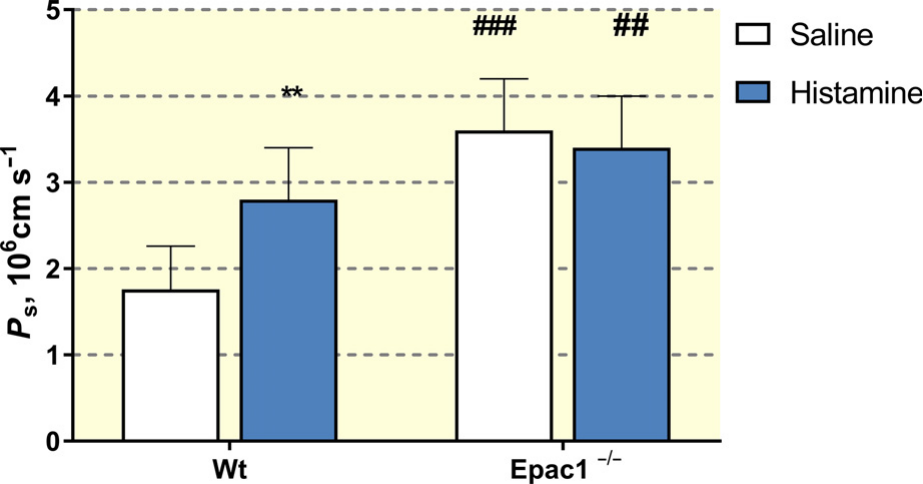
Permeability (*P*_s_) for Gadomer‐17 in wild‐type (wt) mice and Epac1^−/−^ mice in control (i.v. saline) and after histamine administration determined with the two compartment model and the slope/step method. **P < 0.01 for histamine vs control and ##*P* < 0.01 and ###*P* < 0.001 for the same situation comparing within wt and Epac1^−/−^

Reliable values for PS/*V*_a_ for Gadomer‐17 with saline and histamine treatment were obtained from seven of eight wt mice tested. *P*_s_ was estimated assuming *V*_a_/*S* was 4.4 × 10^−4^ as for the Dotarem. Histamine increased mean *P*_s_ for Gadomer‐17 from 1.76 ± 0.5 × 10^−6^ to 2.8 ± 0.6 × 10^−6^ cm s^−1^. The SI curve from the Epac1^−/−^ mice was more variable, and reasonable data from paired experiments with saline and histamine treatment were from 8 of 14 mice. Figure 4 summarizes the results using the compartmental method in the successful experiments. Histamine significantly increased baseline permeability in wt mice. The baseline permeability to Gadomer‐17 was also significantly increased in Epac1^−/−^ mice (3.4 ± 0.6 × 10^−6^), but histamine did not increase permeability further (*P*_s_ = 3.6 ± 0.6 × 10^−6 ^cm s^−1^). Thus, the analysis of Gadomer‐17 data using the compartmental model was also consistent with the observations when using Dotarem and demonstrated that histamine did not increase permeability in Epac1^−/−^ mice above the already elevated (relative to wt mice) baseline permeability.

### 2.6 Response to histamine in wt mice compared to Epac1^−/−^ using Dotarem and the Johnson‐Wilson model

**Figure 5:**
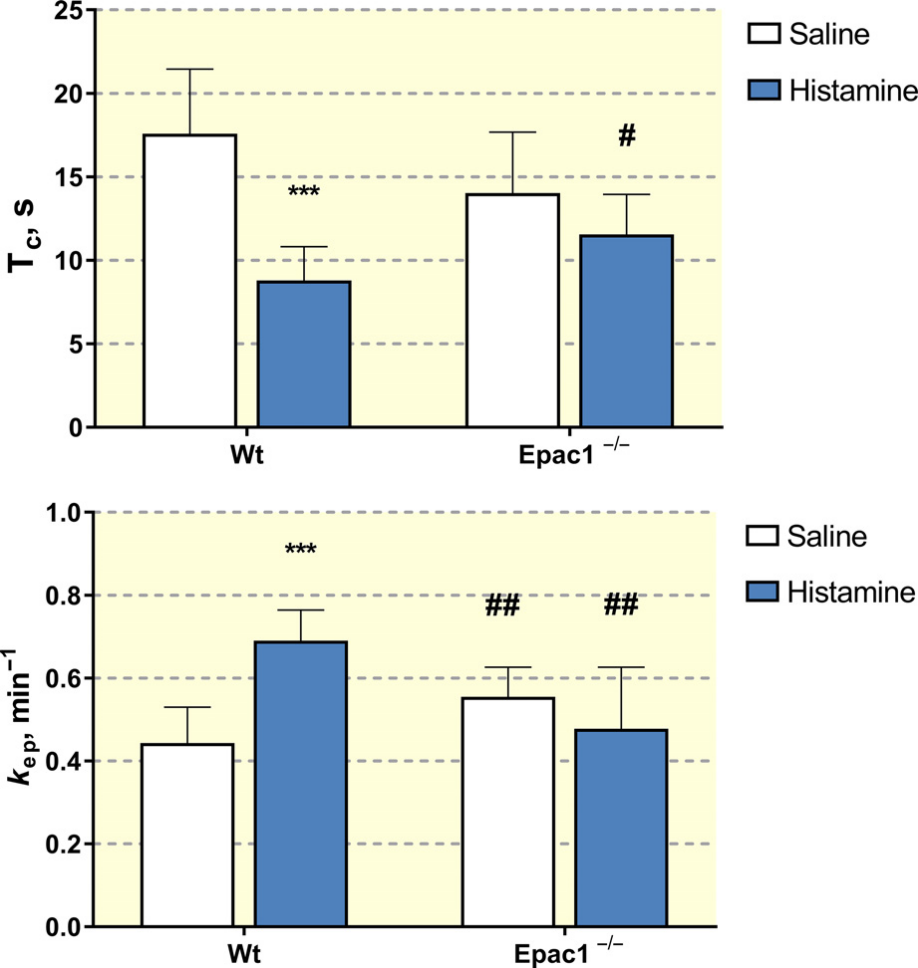
A (Upper panel), Intravascular transit time (*T*_c_) for Dotarem in wild‐type (wt) mice and Epac1^−/−^ mice injected with i.v. saline (control) and after histamine administration. ***P < 0.001 for histamine vs control (saline). ^#^ *P* < 0.05 for the same condition comparing between wt and Epac1^−/−^ (#*P* = 0.052 for control wt vs Epac1^−/−^, *P* = 0.059 for Epac1^−/−^ saline (control) vs histamine). B (Lower panel), Rate constant for transport from the extravascular to the intravascular space (*k*_ep_) for Dotarem in wt mice and Epac1^−/−^ mice injected with i.v. saline (control) and after histamine administration. ***P < 0.001 for histamine vs control and ##*P* < 0.001 for the same situation comparing within wt and Epac1^−/−^

Table 1 summarizes the primary variables *T*_c_, *F*_p_, *E* and *k*_ep_ together with the additional parameters derived from these variables when the Johnson‐Wilson analysis is applied to the same data as in Figure 1. This analysis confirms a significant difference in the response of Epac1^−/−^ mice relative to wt mice after exposure to histamine. Histamine increased Dotarem tracer delivery and tissue uptake in wt mice as indicated by increased blood flow, reduced transvascular transit time (*T*_c_), and increased tissue to plasma exchange measured as the coefficient *k*_ep_. The clear differences between wt mice and Epac1^−/−^ mice are highlighted by comparing transvascular transit times (Figure 5A) and the exchange coefficient (*k*_ep_) (Figure 5B). In contrast to the action of histamine in wt mice (Figure 5A), histamine causes no further increase in the rate constant of tracer exchange, *k*_ep_, in Epac1^−/−^ mice compared with the saline controls in these animals (Figure 5B). Further, *k*_ep_ in the saline controls in Epac1^−/−^ mice are significantly increased relative to the wt controls (0.55 ± 0.07 min^−1^ vs 0.44 ± 0.09 min^−1^, *P* < 0.01). In Epac1^−/−^ mice, there is no significant change in intravascular transit time, *T*_c_, relative to wt controls and *T*_c_ after exposure to histamine, was still 83% of saline control. To summarize, the Johnson‐Wilson deconvolution analysis demonstrated that the saline controls in Epac1^−/−^ mice have an increased tracer exchange (*k*_ep_) and unchanged intravascular transit time (*T*_c_). Histamine failed to modify these parameters in Epac1^−/−^ mice compared to wt mice where *k*_ep_ increased and *T*_c_ decreased.

Although this analysis confirms the failure of Epac1^−/−^ mice to increase exchange above an elevated baseline, the mechanisms contributing to the attenuated response to histamine require an analysis of changes in blood flow and permeability. We note that the JW analysis appears to weight towards increased blood flow. For example the histamine response in wt mice is accounted for by a doubling of plasma flow with no change in PS, but the extraction fractions in tissue (0.71 ± 0.05 vs 0.51 ± 0.08) do not fall as much as expected for no change in PS. Further, while *k*_ep_ is increased in Epac1^−/−^ saline controls relative to wt controls (0.56 ± 0.07 min^−1^ vs 0.44 ± 0.09 min^−1^, *P* < 0.01) this is not accounted for by any change in PS or flow. Finally, the values of blood flow and vascular volume are low compared to published values <10.l mL min^−1^ 100 mL^−1^ vs >20 mL min^−1^ 100 mL^−1^ in several published studies.^8^, ^9^, ^10^ These observations are evaluated further in Discussion.

### 2.7 Dotarem vs Gadomer‐17 in wt mice with Johnson‐Wilson model

As a step towards using Gadomer‐17 as a test tracer in the Johnson‐Wilson analysis, we first carried out paired measurements of both Dotarem and Gadomer‐17 on the same wt mouse (Table 2). The Johnson‐Wilson deconvolution analysis required that the transit time for the two tracers be the same for successive iterations to converge. There are several key observations from the data presented in Table 2. As expected in these wt mice, the estimate of *k*_ep_ for the higher molecular weight tracer Gadomer‐17 was less than that for Dotarem (0.22 ± 0.08 vs 0.61 ± 0.1 min^−1^) and the half‐times for the fall of arterial tracer concentration were nearly 30 times longer for Gadomer‐17 than for Dotarem. Further, the extraction fraction for Gadomer‐17 was <0.1 consistent with significant diffusion‐limited exchange. A striking result was that the estimate of resting blood flow using Gadomer‐17 is more than twice that obtained when using Dotarem (16.7 vs 7.5 mL min^−1^ 100 mL^−1^, *P* < 0.05). The corresponding estimate of local blood volume for Gadomer‐17 was also twice that for Dotarem (2.9 ± 0.8 vs 1.4 ± 0.4 mL 100 ml^−1^, *P* = 0.01). As noted above, both blood flow and local blood volume should be independent of the molecular weight of the tracer and this result points to the Johnson‐Wilson deconvolution model as the source for the discrepancy.

In six additional experiments, the action of histamine on tissue uptake of Gadomer‐17 was tested in wt mice. Histamine significantly increased the plasma flow (from 14.4 ±  to 25.2 mL min^−1^ 100 mL^−1^) and reduced transit times in wt mice. Extraction fraction fell from 0.080 to 0.038. However, there was no significant increase in the model estimates of *k*_ep_, *k*_trans_ or PS. These results indicate either that histamine unexpectedly failed to increase vascular permeability to Gadomer‐17, or that the Johnson‐Wilson model has reduced sensitivity to detect changes in permeability when extraction fractions fell below 0.1. This limitation is supported on theoretical grounds as described in the Discussion. Because of these limitations, the further analysis of Gadomer‐17 tissue SI data using the Johnson‐Wilson model is not reported.

## 3 DISCUSSION

The result that histamine significantly increases vascular exchange in the wt mice, but fails to increase vascular exchange above the already increased baseline level in the Epac1^−/−^ mice is similar to that described in previous experiments using ANP in wt mice and Epac1^−/−^ mice. Taken together these observations conform to the hypothesis that changes in the Epac1 pathways that maintain normal vascular permeability modify the response to inflammatory agents. Before discussing the mechanisms that may underlie these differential responses to histamine of wt and Epac1^−/−^ mice, we evaluate the novel aspects of the present experimental approach. It involves the determination of rate constants for vascular exchange and other key vascular perfusion parameters in paired experiments with and without histamine on the same animal. The present approach using both low molecular weight Dotarem and higher molecular weight Gadomer17 provide new ways to evaluate strengths and weaknesses of analyses of tissue tracer accumulation based on DCE‐MRI imaging approaches. The two most important vascular parameters to be measured to understand vascular exchange are plasma flow (*F*_p_) and PS. The assumptions made in the Johnson‐Wilson model and the simplified two‐compartmental model introduce uncertainties into the estimates of *F*_p_ and PS. We evaluate the issue first with respect to PS.

### 3.1 Johnson‐Wilson model evaluation: evaluation of permeability

Plasma‐to‐tissue exchange is estimated as a flux in the Johnson‐Wilson model and depends on the permeability coefficient of the vascular wall (*P*_s_) the surface area for exchange (*S*) and blood flow (*F*_b_). The model measures only the product of permeability coefficient and surface area which is conventionally written as PS. The data in Figures 3A,B demonstrate that Gadomer‐17 accumulation after histamine administration (Figure 3B) was at least as rapid as the saline control even though arterial tracer concentration was falling more rapidly after histamine i.e. there were smaller plasma‐to‐tissue tracer concentration differences). Under these conditions, the compartmental model (which weighted the whole curve) conservatively described a 50% increase in the rate constants for blood‐to‐tissue exchange with histamine. We can compare this observation with the expected increase in tracer uptake if there was an increase in blood flow with no change in PS. For values of PS/*F*_p_ of the order of 0.1, the fractional increase in tracer uptake calculated as *F*_p_(1−exp−(PS/*F*_p_)) with no change in PS would be <5% increase if flow increased 1.8‐fold as observed (i.e. 25.3 after histamine vs 14.4 mL min^−1^ 100 mL^−1^ in control). Thus, most of the increase in Gadomer‐17 exchange described in Figure 3 was the result of increased *P*_s_. Further, the observation that no increase in PS after histamine was measured using the Johnson‐Wilson model is consistent with the conclusions in the original Johnson‐Wilson paper.^7^ Specifically, Figure 4 of their paper shows that venous tracer concentration (*C*_v_) is very close to *C*_a_ when values of PS/*F*_p_ (α in their paper) are of the order of 0.1 and the difference (*C*_a_−*C*_v_) used to estimate extraction fraction approach zero.

Further, as we noted above, the tissue intensity signals from experiments using histamine and Gadomer‐17 were less stable, and likely added to the problems by introducing significant variability in estimates of the half‐time for the fall in arterial tracer concentration in the AIF and limiting convergence of the deconvolution protocol. Overall, the Johnson‐Wilson model approach is most appropriate for highly diffusible tracers. Most of the detailed analysis of the model in the original Johnson‐Wilson paper focuses on PS/*F*_p_ values much larger than 1 (and in the range 5‐50). In contrast, as noted above, PS/*F*_p_ for Dotarem falls closer to the range when the Johnson‐Wilson model can be more reliably applied (0.4‐1).

### 3.2 Johnson‐Wilson model evaluation: blood flow and blood volume

One clear result was that the measured values of vascular volume and plasma differed significantly between Gadomer‐17 and the lower molecular weight tracer Dotarem. For example, when measured plasma flows are converted to blood flows using a haematocrit of 40%, resting local blood flows estimated using Dotarem in wt mice averaged 7.5 mL min^−1^ 100 mL^−1^ tissue, which is half those estimated from Gadomer‐17 (average 16.6 mL min^−1^ 100 mL^−1^; Table 2). Further, local blood volumes estimated for Dotarem are also around half those estimated using Gadomer‐17 with the respective average numbers of 1.4 compared with 2.9 mL 100 mL^−1^ tissue based on Gadomer‐17 (Table 2). The published values for resting plasma flow in muscle are of the order of 20 mL min^−1^ 100 mL^−1^ or larger^8^, ^9^, ^10^ and the microvascular blood volume expected for a measured surface area of 7 × 10^3 ^cm/100 g tissue for dilated skeletal muscle is greater than 3 mL 100 mL^−1^ (calculated using the same mean volume to surface ratio of 4.4 × 10^−4^ cm in all our calculations above). In previous investigations using radiolabelled albumin as a tracer, we found no difference between the local vascular volumes in muscle tissue in Epac1^−/−^ mice and wt mice.^3^ The most likely reason that Gadomer‐17 appears to provide more reliable estimates of vascular parameters is that it is retained within the vascular space to a larger extent than Dotarem during the measuring period. One example of this is that half‐times for arterial tracer concentration of Gadomer‐17 are several hundred seconds compared to just tens of seconds for Dotarem (see also^11^). From available data on cardiac output and blood volume, the circulation time is between 7 and 10 seconds in a mouse, allowing for blood volume to be circulated for at 6‐10 times in a minute.^4^ With half‐times of the order of 20‐37 seconds for the fall in arterial Dotarem concentration there could be significant loss of the tracer before completely uniform mixing of the Dotarem. Comparable losses with Gadomer‐17 with half‐time more than an order of magnitude greater are much less significant. We note, however we cannot rule a bias introduced into Gadomer‐17 measurement due to the fact that the volume of Gadomer‐17 contrast agent infused was larger than Dotarem (300 vs 120 μL) and the whole vascular volume transiently expanded more in the presence of Gadomer‐17 than Dotarem. It would not be surprising if the combination of slightly increased vascular volume and low extraction fraction also contributed to a bias of the JW analysis towards larger overestimates of blood flow.

### 3.3 Simplified two‐compartmental model: P_s_ and the neglect of flow effect

The compartmental model applied to the Dotarem data also has clear limitations because it does not directly describe changes in plasma flow and concentration gradients along the microvessels are ignored. Nevertheless, the range of uncertainty in the estimation of PS can be calculated for the expected range of PS/F_b_. A key assumption in the simplified two compartment model is that the mean tracer concentration in the exchange microvessels is equal to the arterial concentration. This is, however, not valid when there is an arterial to venous gradient of concentration along the exchange vessels. Failure to account for the gradient of tracer concentration along the blood vessels would lead to an underestimate of *P*_s_. For Dotarem, the worst case is to use values based on the low estimates of F_b_ in wt mice from Table 1, which gives PS/*F*_p_ close to 1 in control for wt mice. In this situation, venous concentration would be as low as 37% of the arterial concentration and a mean concentration in the exchange vessel close to 70% of arterial concentration. Thus, *P*_s_ could be underestimated by about 30%. However, if blood flows were twofold larger (as measured with Gadomer‐17), the underestimate would be closer to 20%.

An additional assumption in the compartmental model is that permeability coefficients (Figures 2 and 4) can be calculated from the rate constants (PS/*V*_a_), assuming a reasonable value for *V*_a_/*S* which is also unchanged even when blood flow increases. The latter assumption is valid, for example, if histamine increased the number of exchange microvessels that were perfused, but the distribution of the increased blood flows between small and larger microvessels after histamine was the same as saline control. On the other hand, if histamine‐induced vasodilation increased surface area for exchange (*S*) more than it increases local vascular volume (*V*_a_), *V*_a_/*S* after histamine should be smaller, and the use of a constant value of *V*_a_/*S* would overestimate *P*_s_ after histamine relative to controls. This would be the case if histamine‐induced vasodilation results in more small diameter capillaries being perfused compared to the number of perfused venular microvessels. For example, if the surface area of perfused capillaries doubled relative to venular vessels, V_a_/S would be reduced from 4.4 × 10^−4^ cm to close to 3.3 × 10^−4^ cm and P_s_ would be overestimated by close to 33%. Thus, likely errors in the compartmental model for Dotarem range from an underestimate of close to 30% (due to arterio‐venous gradients) to an overestimate of a similar magnitude due to redistribution of flow. If both sources of error were present, they may cancel each other.

Finally, we can use the same approach as described above using Gadomer‐17 to evaluate the contributions of increased blood flow and increased PS to the histamine response with Dotarem. For PS/*F*_b_ close to 0.5 (a maximum value from Table 1 in the presence of histamine), increased blood flow with no increase in the value of PS would account for about 25% of tissue uptake. This is less than the twofold increase in tracer uptake of Dotarem shown in Figure 1. Thus, we conclude that both diffusion and flow contribute significantly to tissue uptake of Dotarem.

### 3.4 Mechanisms of action of histamine in wild type vs Epac1^−/−^ mice

The difference between wt and Epac1^−/−^ mice may be understood in terms of the action of how Epac1 pathways maintain normal permeability in all microvessels (capillaries and venular microvessels) by stabilizing cell‐cell adhesion.^1^, ^2^ We have already shown that Epac1 pathways maintain the in vivo basal state permeability and the density of endothelial cell adhesions in the microvessels of major tissues like skin and skeletal muscle.^3^ An important permeability‐promoting action of histamine is to increase the tension within the endothelial cells forming the walls of venular microvessels.^2^, ^12^ We expected, considering the weaker intercellular adhesion in the capillary wall of the Epac1‐deficient mice, that histamine would increase the permeability of Epac1^−/−^ endothelium beyond that observed in wt mice in the basal state. The present study showed, contrary to this expectation, that histamine did not increase further the microvascular passage of MRI‐active probes in the Epac1^−/−^ mice.

In fact, histamine alone led to a similar increase in microvascular permeability as Epac1 deletion, but had no additional effect in the Epac1^−/−^ microvasculature. It appears therefore that the elevated baseline permeability of masseter muscle Epac1^−/−^ microvessels was already near the maximum induced by histamine. A strong cAMP stimulation can blunt the permeability‐enhancing effect of histamine in some microvascular systems.^12^, ^13^ This suggests that histamine and cAMP may act via opposing mechanisms. A recent study implicates the adherens junctions as being pivotal for the histamine‐induced tension of endothelial cells. The proposed mechanism is that the fibres induced to contract by histamine require anchoring to intact adherens junctions to achieve widening of the intercellular cleft.^14^ A working hypothesis that may explain previous observations by others as well as the present findings is therefore that histamine acts to widen the clefts between endothelial cells mainly when they have near basal cAMP/Epac1 stimulation. In the absence of Epac1, the contractile fibres stimulated by histamine may lack strong anchoring to adherent junctions. On the other hand, at very high Epac1 (and PKA) activation, the adherens junctions may be too tight for the histamine‐induced contractile fibres to widen substantially the intercellular cleft. We note that our previous observation that ANP fails to increase permeability above elevated baseline values suggests that changes to the tethering of molecular complexes at adherens junctions of Epac1^−/−^ mice are likely to account for our observations with both histamine and ANP.

### 3.5 Further applications

On a more technical note, the study demonstrates that paired sequential experiments using more than one tracer in each animal combined with analytical methods provide a more internally consistent description of modulations of microvascular function than experiments using only one tracer and unpaired experimental design. The study also demonstrated that the numerical analysis of DCE‐MRI data with tracers of different molecular weight has significant limitations.

Finally, we note that the ongoing development and evaluation of methods to apply DCE‐MRI imaging to the evaluation of microvascular functions focuses not only on muscle tissue in mouse models as described here, but also transvascular exchange in tissue with widely different permeability and blood flows. A recent study used DCE‐MRI and Dotarem to study the blood‐brain barrier in patients and described several different models including versions of the compartmental model used above for fitting the MRI data.^15^ The conclusion was that the Patlak model accounting for simultaneous diffusion and convection best fitted their observations on the human blood‐brain where the vascular permeability coefficients are up to two orders of magnitude smaller than muscle and where the human circulatory system is less hyperdynamic compared to mice (e.g. heart rate up to 600 beats/min). On the other hand, an important area for both experimental and clinical investigation is the tumour vasculature. There is evidence that altered microvascular permeability is an early sign of neoplastic growth which correlates with the degree of malignancy of solid tumours.^16^, ^17^, ^18^ In tumours, a wider range of plasma flows, vascular permeability and high levels of vascular heterogeneity than encountered in the present study is likely. The limitations for the use of DCE‐MRI as demonstrated for masseter muscle in this study may not be the same for such different applications. Nevertheless, the careful evaluation of the tissue SI curves using separate tracers and complementary analyses as illustrated using Gadomer‐17 and Dotarem are likely to provide more reliable information that studies based on a single tracer and one method of analysis. An important message from this study is that the tracer used for DCE‐MRI must be tailored towards the permeability characteristics of the tissue and vascular bed that is to be studied.

## 4 MATERIALS AND METHODS

### 4.1 Ethical approval

Animal experiments were approved by the Norwegian Animal Research Authority (approval number 4894). The study was conducted according to the European Convention for the Protection of Vertebrates Used for Scientific Purposes, Norway. The study was performed at the Department of Biomedicine and the Molecular Imaging Facility at the University of Bergen, Norway.

### 4.2 Animal experiments

#### 4.2.1 Wild type and Epac1^−/−^ mice

Generation of Epac1^−/−^ mice has been described in detail elsewhere.^3^ Briefly, heterozygous floxed Rapgef3 mice were generated at the Mouse Clinical Institute, Strasbourg, France. They were subsequently crossed with C57BL/6J mice expressing CRE recombinase from the CMV promoter resulting in global deletion of Epac1 expression, as confirmed by immunoblotting and RT‐PCR.^3^ C57BL/6JBomTac mice from Taconic, Denmark, were used to backcross the recombined chimeric mice for at least 10 generations.^3^ C57Black were used as controls for the Epac1^−/−^ mice.

#### 4.2.2 General

The animals were kept with artificial lighting on a 12:12‐hour light‐dark cycle. Room temperature was kept constant at 23°C, and the animals were provided with water and rodent chow ad libitum. The mice were 8‐10 weeks old when used for experiments. The weight range was 19‐21 g and an equal number of male and female mice were used. All experimental procedures were performed while animals were anaesthetized as described below. A single i.v. catheter (polyethylene, i.d. 0.28 mm and o.d. 0.61 mm, Portex, Smiths Medical International Ltd, Kent, UK) was connected to a 30G insulin syringe (Omnican, B. Braun Melsungen, Melsungen, Germany) and placed in a tail vein.

#### 4.2.3 Dynamic‐contrast enhanced magnetic resonance imaging of the masseter muscle

During scanning, the mice were anaesthetized using 3.0 ± 1.0% sevoflurane mixed with oxygen at a flowing rate of 200 cc/min. Respiration rate and body temperature were monitored using MRI‐compatible animal monitoring equipment (SA Instruments, Stony Brook, NY, USA). Respiration averaged around 60‐80 cycles/min, but increased, sometimes as much as twofold, immediately after the injection of histamine. The core body temperature was kept at 35 ± 1°C. The depth of anaesthesia was evaluated by toe pinch prior to starting placing the intravenous catheter in the tail and from the respiration frequency (60‐80 per min) during scanning. The time required for the scanning was 25 min. During the first scanning session, the mice received saline i.v. After the scanning, the i.v. line was removed and the mice woke up. The next day they were re‐anaesthetized and a new venous catheter was introduced in the tail vein. The mice then received histamine instead of saline and the scanning was repeated with the same parameter settings as the first day. At the end of the procedure and while still anaesthetized, the mice were killed by breaking their stretched neck with a blunt stroke with a pair of scissors.

The MRI experiments were performed on a 7T horizontal‐bore preclinical scanner (Pharmascan 70/16; Bruker Corporation, Karlsruhe, Germany), using a 23 mm ID mouse‐head quadrature volume resonator in a single‐coil (TX/RX) configuration. Estimation of microvascular haemodynamic parameters was based on a 2D FLASH acquisition with a 17‐degree flip angle and 1400 image frames with a time resolution of 1.080 seconds. The total scan time was therefore 25 minutes 12 seconds. In addition, T1 mapping was performed before contrast injection using the same acquisition (2D FLASH) and a range of flip angles (3^°^, 5^°^, 7^°^, 10^°^, 15^°^, 17^°^, 24^°^, 31^°^). The MRI parameters for the two sequences were as follows: *TE*/*TR* = 2.10/15.00 ms, 1 average, matrix = 96 × 96, FOV = 25 × 25 mm^2^, resolution = 260 × 260 μm^2^, slice thickness = 75 μm. Two slices were acquired, one through the masseter muscle and one along the external jugular vein for assessment of arterial input function.

#### 4.2.4 Investigation of the effect of histamine

The animals received saline on the first day and histamine on the following day as a volume of 150 μL of either saline (control) or histamine (dose 0.2 mmol/kg body weight BW)^19^ into the tail vein during 10 seconds using an injection pump. Although starting with the dosage given by^19^, we had to reduce it to 0.2 mmol/kg which was the highest tolerated by the mice used in this study. Each animal was imaged either with Dotarem (Gd‐DOTA, molecular weight 0.56 kDa) or Gadomer‐17 contrast agent (molecular weight 17 kDa, but with Stokes radius corresponding to a globular protein of molecular weight 30‐35 kDa). Both contrast agents were injected at 0.44 mmol Gd/kg bw. Dotarem was injected in a mean volume of 120 μL. In order to achieve the gadolinium dose for Gadomer, it had to be injected in an average volume of for and Gadomer‐17 in a mean volume of 300 μL. The contrast agent was injected 1.5 minutes after the injection of saline or histamine via the mouse tail vein over a period of 15 seconds using an MRI‐compatible programmable injection pump (Harvard Apparatus, Massachusetts, USA). The control experiments with saline were performed 1 day before the experiments with histamine.

### 4.3 Experimental groups

#### 4.3.1 Effect of histamine in wild type mice

Seven mice received saline followed by Dotarem 1.5 minutes later and MRI signals were recorded as described above. The i.v. catheter was removed and the mice woke up within minutes after cessation anaesthesia. On the subsequent day, the same seven mice received first histamine and then Dotarem 1.5 minutes later with MRI recording as described above. After this recording and while still anaesthetized, the mice were killed by breaking their stretched neck with a blunt stroke with a pair of scissors. Eight other mice underwent the same procedure but received Gadomer‐17 instead of Dotarem.

#### 4.3.2 Effect of histamine in Epac1^−/−^ mice

The procedures were exactly as described above for wt mice. On day one for the Dotarem studies, 10 Epac1^−/−^ mice received saline followed by Dotarem 1.5 minutes later and the MRI signal was recorded as described. The i.v. catheter was removed and anaesthesia was terminated and the mice woke up within minutes. On the subsequent day, the same mice received histamine prior to Dotarem and the MRI signal was recorded as described. After the recording and while still anaesthetized, the mice were killed as described above. Twelve other mice underwent the same procedure as the first 10 mice, but received Gadomer‐17 instead of Dotarem.

#### 4.3.3 Comparison of Dotarem and Gadomer‐17 in the same mouse

Six mice received saline followed by Dotarem 1.5 minutes later and the MRI signal was recorded as described. The i.v. catheter was removed and the mice woke up within minutes after cessation of anaesthesia. On the following day, Gadomer‐17 was given after saline as described and recording of the MRI signal started 1.5 minutes later. The mice were killed while still anaesthetized as described above.

### 4.4 Numerical models for analysis of the MRI signal

#### 4.4.1 Model parameters

In previous studies, we used a modified two‐compartmental model to extract estimates of the rate constant for blood‐to‐tissue exchange (PS/*V*_a_) for Gadomer‐17 in mice exposed to ANP.^3^, ^5^ PS is the permeability‐surface area product and *V*_a_ is the local microvascular volume. This model makes simplifying assumptions about the ratio (*V*_a_/*S*) and gradients of tracer concentration across a microvascular bed to estimate permeability coefficients during vascular perfusion. These assumptions may be compromised when more permeable lower molecular weight tracers such as Dotarem are used or when both permeability and blood flow are increased by an inflammatory agent.

We therefore also tested an alternate approach based on the classical Kety/Renkin/Crone model of tracer extraction in a perfused microvessel. The extraction fraction (*E*) is defined as (*C*_a_−*C*_v_)/*C*_a_ where the *C*_a_ is the arterial tracer concentration and *C*_v_ is the tracer on the venular side of the microvasculature bed. *E* is determined by both plasma flow (*F*_p_) and the product of permeability coefficient *P* and surface area (*S*) product:

(1) $$E=1-\exp(-\text{PS}/F_{p})$$

The Johnson‐Wilson form of this model requires high extraction fraction of the tracer and rapid tracer equilibration in the extravascular space. It takes into account the volume (*V*_e_) of the extracellular space available to the tracer and exchange between the tissue and the plasma is described in terms of exchange rate constant *k*_ep._ 7

(2) $$K_{\mathrm{ep}}={\text{EF}}_{b}/V_{e}$$

In previous publications, detailed evaluations of deconvolution methods (advanced curved fitting) have been described to evaluate the Johnson‐Wilson model and tested using Dotarem as a tracer. The approach enables estimation of four model parameters that describe tissue uptake: The transvascular transit time *T*_c_, plasma flow rate *F*_p_ as well as *E* and *k*_ep._ 20, 21, 22 Blood flow is calculated as *F*p/(1‐Hct) where Hct was set at 40%. The parameters, permeability‐surface area product, PS, local vascular volume for the tracer in a region‐of‐interest (ROI) (*V*_a_), extracellular volume available to the tracer (*V*_e_) and a tracer clearance term *k*_trans_ are then derived from these estimates. The increase in the number of parameters in the Johnson‐Wilson model analysis introduces additional parameter sensitivities to both noise in the data and signal strength when physiological changes are relatively small.

#### 4.4.2 Deconvolution methods

The analysis of the time course of the SI curves from MRI experiments in mice using an arterial input function (AIF) and a tissue residue function (TRF) was derived from the Johnson‐Wilson exchange model.^7^, ^20^, ^21^, ^22^ Briefly, the analysis describes the observed sequence of the DCE‐MRI tissue signal intensity (*S*_t_) in terms of n iteration steps where *S*_t_(*n*) = *C*_p_ (*n*)*H(*n*). Here, *C*_p_(*n*) is the AIF and *H*(*n*) is the TRF multiplied by the *F*_b_. Successive updates of both the TRF and then the AIF functions are made until convergence as described.^22^

For the JW model, the vascular phase is

$$H\left(n\right)=F_{p}<nT_{s}<T_{c}$$

where *T*_s_ is the sampling interval and *T*_c_ is the mean intravascular transit time.

The tissue phase is

(3) $$H\left(n\right)=V_{e}k_{\mathrm{ep}}\exp\left(-k_{\mathrm{ep}}nT_{s}\right),nT_{s}>T_{c}$$

From the analysis,^20^ values of *k*_ep_, *E* and *F*_p_ are obtained for a user defined range of *T*_c_.

Previous evaluations of the method have demonstrated that attempts to obtain an AIF from measurements of the changes in SI of a tracer over a small artery separate from the signal over the tissue were not successful in the mouse, whereas an AIF determined from the so‐called blind deconvolution approach converges. A particular advantage of the blind deconvolution method is that only the integral of the AIF is needed for the analysis. This reduces the dependence on noise due to respiratory and vascular disturbances on the AIF. However, the evaluation of this integral from the area under the SI measured over the tissue requires an independent estimate of the volume of distribution of the tracer in the vascular and extravascular compartments in a given organ. For a molecule of the size and properties of Dotarem, the distribution volume is about 0.12 mL mL^−1^ of muscle tissue.^22^, ^23^ No independent measurement of this distribution volume is available for Gadomer‐17, but its apparent molecular weight would indicate that it is similar to that of albumin (cf, ref. 23). Because of its high molecular weight and low permeability in muscle tissue, Gadomer‐17 tracer distribution is biased towards the vascular volume.

#### 4.4.3 Analysis based on two compartment model

The compartmental model is developed from the so‐called step‐slope analysis. The analysis was first developed for fluorescently labelled tracers in individually perfused microvessels^24^ and later modified for fluorescent and MRI measurements in mice.^5^, ^25^

The simplest analysis of the SI vs time curve is based on the assumption that, after filling the vasculature, tracer accumulation is in the extravascular space and is driven by the concentration difference of tracer between plasma and interstitial fluid.

Tracer accumulation in the extravascular volume *V*_e_ was described by Ficks Law:

(4) $$V_{t}\left(dC_{t}/d_{t}\right)=\text{PS}\left(C_{a,\mathrm{mean}}-C_{t,\mathrm{mean}}\right)$$

where *C*_a, mean_ and *C*_t, mean_ are the average tracer concentration on the vascular and extravascular compartments.

The fall in *C*_a_(*t*) with time was approximated by a first‐order fall in tracer concentration towards a quasi‐equilibrium in the body described by the relation:

(5) $$\left(C\left({}_{t}\right)-C_{\mathrm{equil}}\right)/\left(C_{0}-C_{\mathrm{equil}}\right)-\exp\left(-k_{\mathrm{art}}t\right)$$

where *C*_equil_ = 0.27 *C*_o_ assuming a whole body the extravascular space that was 2.7 times larger than the vascular space and the combined distribution space is 3.7 times the initial distribution space and *k*_art_ is the rate constant. We tried to derive initial estimates of *k*_art_ for Dotarem from the SI measured over an adjacent blood vessel flowing near the measuring window within the masseter muscle but, as with the deconvolution analysis, this approach was not successful because the values were quite variable and the estimated values were too large to account for the initial rate of fall SI intensity over the measuring window after the step increase in signal intensity. The values of *k*_art_ are therefore adjusted independently to fit data for each animal.

For Dotarem, which was expected to be filtered freely in the kidney, an additional term was needed to describe the continued fall in arterial tracer concentration after the initial rapid distribution of the tracer towards a quasi‐equilibrium between plasma and body tissues. This was set equal to the first‐order time constant equal to GFR divided by whole body plasma volume. Unpublished measurements of mean GFR in these mice from one of our labs (SOD) are 220 μL min^−1^ in wt mice and 275 μL min^−1^ in Epac1^−/−^ or 7.3 and 9.2 μL min/g BW respectively. Whole body plasma volumes are 30‐35 μL/g BW for both wt and Epac1^−/−^ mice, giving rate constant for renal excretion of Dotarem 0.0036 s^−1^ and 0.0046 s^−1^ for wt and Epac1^−/−^ mice respectively. Thus, for Dotarem, the fall in arterial concentration at each interval in time in wt mice was calculated from Equation (4) with the additional correction for renal excretion with a time constant of −0.0036 s^−1^.

The accumulation was calculated by numerical integration where total accumulation relative to the initial amount equals (*V*_a_ *C*_a_ (*t*) + *V*_e_ *C*_t_)/*V*_a_ *C*_t_ (_0_).

For completeness, the rate constant for renal or other non‐specific Gadomer‐17 loss was arbitrarily set at 5% of the Dotarem renal excretion rate. The compartmental model is similar to the diffusion‐limited compartmental model used in previous analyses.^26^

### 4.5 Statistical analysis

Data are presented as mean ± 1 SD. Statistical significance within each experimental group (Groups A, B and C) was investigated using two‐tailed Student's *t* test for paired comparison when analysing the effect of i.v. histamine vs i.v. saline as well as in Group C when comparing Dotarem and Gadomer‐17 in the same mouse. Comparison between wt vs Epac1^−/−^ (Groups A and B) was done with two‐tailed t test for independent groups. *P* < 0.05 was considered statistically significant.

## CONFLICTS OF INTEREST

None.

## AUTHOR CONTRIBUTION

FEC and RKR conceived the study and wrote manuscript, SOD generated Epac^−/−^, SOD and RB tested and selected mice suitable for the experiments, CBR and TP performed the experiments, CBR, TP, TT, FEC and RKR analysed data, all the authors read, commented upon and approved final version of the manuscript before submission.
